# MALDI MSI Separation of Same Donor’s Fingermarks Based on Time of Deposition—A Proof-of-Concept Study

**DOI:** 10.3390/molecules28062763

**Published:** 2023-03-19

**Authors:** Kim Frisch, Kristine Lykke Nielsen, Simona Francese

**Affiliations:** 1 Department of Forensic Medicine, Aarhus University, 8200 Aarhus N, Denmark; 2 Centre for Mass Spectrometry Imaging, Biomolecular Sciences Research Centre, College of Health, Wellbeing and Life Science, Sheffield Hallam University, Sheffield S1 1WB, UK

**Keywords:** MALDI MSI, overlapping, fingermarks, age

## Abstract

Despite the advent of DNA profiling, fingerprints still play an important role in suspect identification. However, if single crime scene marks may be challenging to identify, overlapping fingermarks, understandably, pose an even greater challenge. In the last decade, mass spectrometry-imaging methods have provided a possible solution to the separation of fingermarks from two or more donors, based on the differential chemical composition. However, there are no studies attempting to separate overlapping marks from the same donor. This is important in relation to fingermark deposition at different times, which could be critical, for example, to ascertain legitimate access to the scene. In the work presented here, we investigate whether Matrix-Assisted Laser Desorption Ionisation Mass Spectrometry Imaging can separate the same donor’s fingermarks deposited at different times based on intra-donor fingermark composition variability. Additionally, the hypothesis that the different times of deposition could be also determined was investigated in the view of linking the suspect at the scene at different times; the dating window of MALDI MSI within the selected molecular range was explored. Results show that it is possible to separate overlapping fingermarks from the same donor in most cases, even from natural marks. Fresh marks (0 days) could be separated from those of fourteen days of age, though the latter could not be distinguished from the set aged for seven days. Due to the use of only one donor, these are to be considered preliminary data, though findings are interesting enough to warrant further investigation of the capabilities and limitations of this approach using a larger cohort of donors.

## 1. Introduction

Despite the advent of DNA profiling, fingerprints remain a primary form of biometric identification informing both investigations and judicial debates. A suspect identification is typically made by comparing a crime scene mark to a fingerprint record held in national databases (provided that the suspect has had a previous arrest) or to a fingerprint taken from a person of interest. Whilst three levels of details are available to fingerprint experts to compare a crime scene mark to a fingerprint, the composition and distribution of local characteristics of the ridge pattern called *minutiae* (level 2 details) are particularly important for identification, after having ascertained that the less specific level 1 details are present (e.g., arch, loop, delta etc.). Whilst generally successful, fingerprinting becomes difficult for smudged, faint, and very partial marks. Additionally, overlapping fingermarks pose a further challenge given by the difficulty to distinguish two or more ridge patterns to assign these impressions to the individuals that have generated them. The ability to separate overlapping fingermarks would be very important for both eliminating irrelevant marks and distinguishing, for example, between the victim’s and the offender’s fingermarks. In the last 15 years, the analytical community has offered spectroscopic imaging [1,2,3] and mass spectrometric imaging solutions to this problem (in combination with various chemometric approaches) [4,5,6,7,8,9,10]. All the approaches proposed are based on the principle that, as hundreds to thousands of molecules can be detected in fingermarks and as this composition varies all the time, it is possible to pick out those compounds that are uniquely present in each of the marks analysed and can be selected to reconstruct the fingermark ridge pattern. In terms of the mass spectrometric approach, a few techniques have been reported to successfully separate fingermark impressions, namely Desorption Electrospray Ionisation Mass Spectrometry imaging (DESI MSI) [4], Matrix-Sssisted Laser Desorption Ionisation Mass Spectrometry Imaging (MALDI MSI) [5,6,7], (nanoparticle-assisted) Laser Desorption Ionisation [8,9] and time-of-flight Secondary Ion Mass Spectrometry (ToF-SIMS) [10]. 

Whilst these techniques have been used to separate ridge patterns from two or more individuals, to the authors’ knowledge, no study has been reported to date attempting separation of fingermarks left on a surface by the same individual.

The only studies that are relevant to this subject are from Lauzon et al. [11] and Gorka et al. [12,13]. In their report [11], the former group suggests the possibility to use the chemical profile of the fingermark composition to connect fingermarks of the same individual left in different areas of a crime scene. For example, should a smudged mark be unidentifiable in one area of the crime scene (for example the kitchen), it could be assigned to a certain individual, should their identifiable mark be found there or in a different area (for example the living room). This link would provide the opportunity to reconstruct the movements of the offender during the crime based on the principle that the molecular profiles of the smudged and identifiable marks are the same. This assumption would be much easier to accept for marks that have been deposited at the same time. However, despite the long-standing common understanding and literature-supported knowledge that the fingermark composition varies according to metabolism, pharmacological and pathological states, stress, physical activity, diet, etc., Becue’s group has been challenging the fingerprint community [12,13] with the idea that the intra-donor fingermark chemical composition remains fairly constant over time; as a result, it is potentially distinctive of an individual (some sort of biological passport) or permits categorisation of a group of individuals. In their recent MALDI MS-based work, Gorka et al. [13] analysed natural fingerprints from 13 individuals over 12 months, concluding that overall “the composition of the fingermarks provided by the 13 donors is quite consistent over the months and the year”. Specifically, they report that *(i)* the compositions at different times of the year (for example January vs. July) are closely linked in the heatmaps and *(ii)* the compositional consistency observed over the year is also reproducible between the thirteen donors given that percentage of the *m*/*z* features retained between each selection is similar for every participant to the study. Whilst the heatmaps are sound, donors’ habits, medications, diet, etc. at the time of each fingermark deposition does not appear to have been recorded. Additionally, donors were only asked to not wash their hands 45 min prior to each deposition and thus reducing the chance of picking up different contaminants. Importantly, whilst the composition may be constant, the abundance of those components may be different, and the resulting ion intensity should be considered a discriminant of the intra-donor chemical profile. Finally, due to lower mass range chosen (100–2000 Da) and the fact that only some species may ionise in positive mode or are not masked by the presence of the matrix selected, the vast majority of the anti-microbial peptides and small proteins [14] as well as amino acids are excluded from detection. Therefore, whilst certainly a very interesting paper, these uncertainties still justify the investigation into the separation of overlapping fingermarks deposited by the same individual at different times by MALDI MSI. The possibility to separate these types of marks is extremely relevant in a forensic context as it may inform on the legitimate access or lack thereof of the suspect to the scene. If same-day fingermark compositions cannot be distinguished, their separation must mean they were deposited at different times, whilst linking the suspect to the scene (through the provision of biometric identification). There remains the issue of dating a crime fingermark with some notable efforts from the mass spectrometry community [15,16,17,18]. The issue of establishing the age of a fingermark is not extensively addressed here as it needs, in the authors’ opinion, a much larger, concerted, and systematic approach to be appropriately tackled. However, a proof-of-concept verification of the hypothesis that it is possible to separate the same person’s fingermarks deposited at different times will both contribute to add complementary knowledge to that provided by Gorka et al. [13] and to the global fingermark-dating problem. Results from the present study have shown it is possible to separate overlapping fingermarks from the same donor in most cases, although it becomes more difficult to observe *minutiae* for some marks older than 15 days (often due to sub-optimal contact pressure resulting in ridge merging). As only one donor was used, capabilities and limitations will need to be verified with a larger cohort of donors.

## 2. Results and Discussion

The separation of overlapping fingermarks and fingermark age determination by mass spectrometry-based approaches are topics that have been previously investigated and reported in the scientific literature [4,5,6,7,8,9,10]. However, to date, no study has been published on the separation of *overlapping fingermarks* from the *same donor* and *deposited at different times*. 

Preliminarily, groomed fingermarks were analysed to address the hypothesis that the molecular composition of fingermarks from the same donor, but of different ages, is different enough to permit separation of the ridge pattern. Partially overlapping groomed fingermarks of different ages were therefore generated by the same donor in a total of eight pairs, as shown in Table 1.

**Figure 1:**
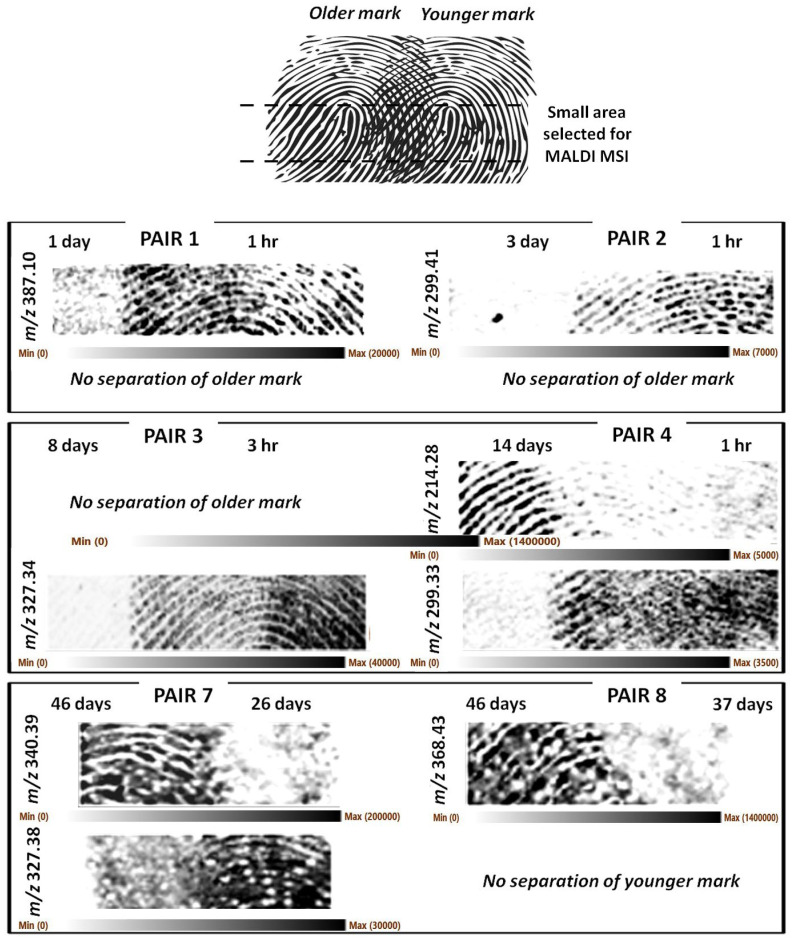
MALDI MS images of six pairs of partially overlapping same-donor groomed fingermarks on aluminium slides. The fingermarks were aged for the times indicated in the figure and in Table 1. Top part of the figure illustrates the schematics of the pair arrangements. Pairs 5 and 6 reported in Table 1 did not yield any suitable fingermark images and were not shown. Older marks could be separated for pairs 4 and 7. Younger marks could be separated for all the pairs shown except for pair 8. MALDI MSI images have been obtained at 100 µm × 100 µm spatial resolution.

Only a small section of the mark was imaged to reduce the run time and ensure all the intended analyses could be completed in the time available for this study. Figure 1 shows representative *m*/*z* ions responsible for the separation of the marks. 

As it can be observed, it was generally possible to achieve separation of the marks albeit with some exceptions. These exceptions were mainly due to occasional lack of clarity of the ridge detail, likely resulting from a combination of sub-optimal matrix application and occasional sub-optimal fingermark deposition leading to ridge merging due to excessive contact pressure. The latter circumstance was particularly deleterious for pairs 5 and 6, which did not generate any useful images and were not reported in Figure 1. Figure S1 reports the optical images of the groomed set showing instances of sub-optimal fingermark deposition. 

In other circumstances, the image of the younger fingermark could be separated/recovered but not that of the older mark (pair 1- 1 d vs. 1 h, pair 2- 3 d vs. 1 h, pair 3- 8 d vs. 3 h). However, this circumstance does not mean fingermarks as old as 1 day and older cannot be imaged; in fact, pairs 4 and 8 have yielded images of 14- and 46-day-old marks. In one case only, it was the younger (37 d) fingermark image that could not be separated/recovered (pair 8- 46 d vs. 37 d). 

It was reasonable to hypothesise that for groomed marks, endogenous species would be the main separating ions, given that marks were generated by rubbing the fingertips on the forehead and cheeks prior to subsequent deposition of the marks. However, it is important to note that for the scope of this paper, which is the attainment of biometric information by separating two impressions, knowing the identity of the ions that separate the two fingermarks is not essential. Whichever is the ion fit for this purpose, in any given instance, can be used without further investigating its identity. In any case, we have attempted a putative identification of the separating ions. It is likely the ions at *m*/*z* 340.394 and 368.426 are exogenous quaternary ammonium ions (C_23_H_50_N^+^, mass accuracy −2.3 ppm and C_25_H_54_N^+^, mass accuracy −3.8 ppm, respectively). While the ion *m*/*z* 327.337 (separating the younger mark of pair 3) could not be putatively identified, the ion at *m*/*z* 327.382 (partially separating the younger mark of pair 7) may be the C-13 isotope of the didecyldimethylammonium ion discussed later in this paper. As for the other ions separating the fingermarks, a quick lipid map search (https://www.lipidmaps.org, accessed on 17 March 2023) using a mass tolerance of +/−0.001 Th yielded a putative identification only for the ion at *m*/*z* 387.099 as a potassiated phospholipid derivative, lysophosphatidic acid LPA 12:3 of formula C_15_H_25_O_7_PK (mass accuracy −5.7 ppm), reported as involved to train the skin to repair and strengthen the epidermis [19]. 

To investigate applicability in real case scenarios, these analyses were repeated using natural marks, which are impressions made without grooming or any prior preparation of the fingertip. However, the marks were aged for slightly different duration with respect to the groomed marks (Table 1), generating another set of eight pairs of partially overlapping fingermarks (Table 2), though employing the same donor and environmental aging conditions. 

The slight difference in the age of the marks was dictated by instrumental availability. This natural mark set was then analysed by MALDI MSI upon optimisation of the matrix deposition conditions that are reported in the Methods Section 3.2.5.

**Figure 2:**
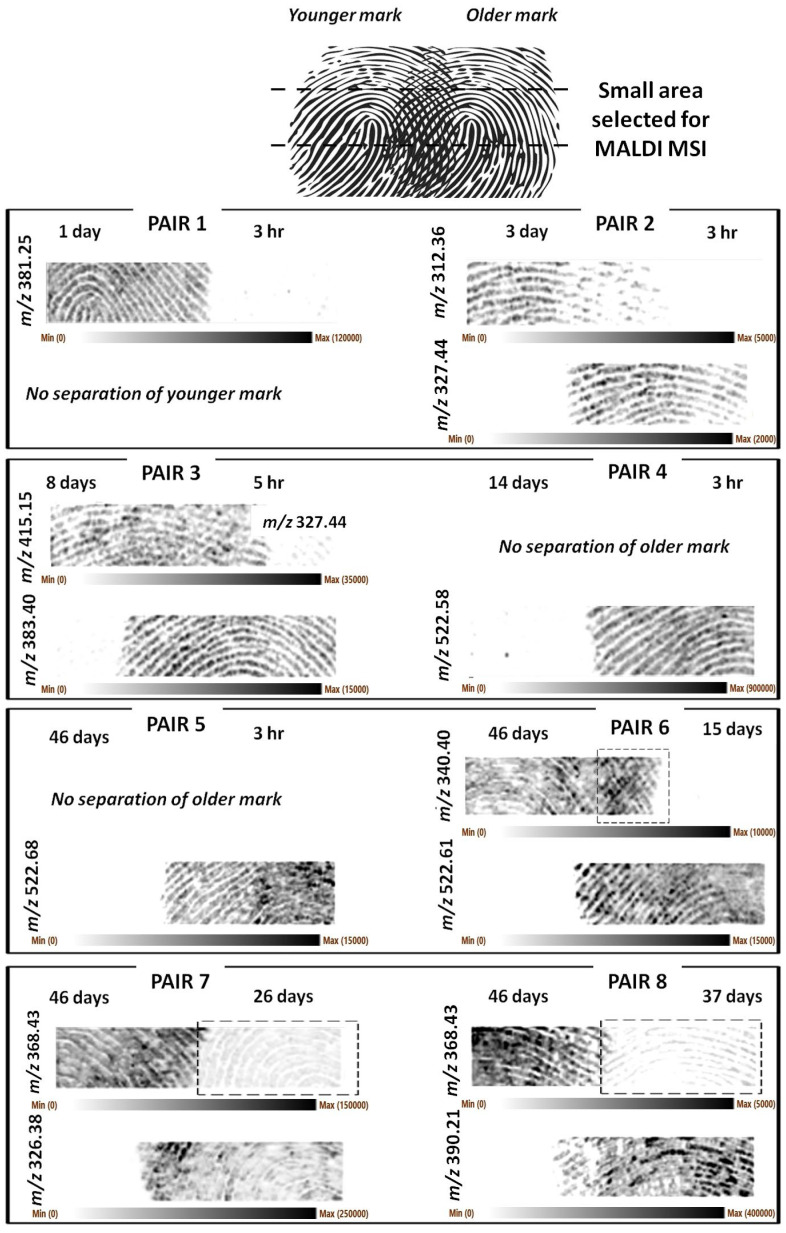
MALDI MS images of eight pairs of partially overlapping natural fingermarks deposited on aluminium slides. Top part of the figure illustrates the schematics of the pair arrangements. The fingermarks were deposited by the same person and aged for the times indicated in the figure and in Table 2. Older marks could be separated for pairs 2–3, 6–8. Younger marks could be separated for all the pairs shown except for pair 1. The squared frame in pair 6 indicates part of the younger mark has not been resolved. The squared frames from pairs 7 and 8 indicate the *m*/*z* ion used to obtain the images of the older marks is also less abundantly present in the younger marks. MALDI MSI images have been obtained at 100 µm × 100 µm spatial resolution.

In this case too, Figure 2 shows representative *m*/*z* ions responsible for the separation of the marks. 

A higher image resolution and clarity of the fingermark ridge detail was achieved in this instance. However, it was clear that for natural marks too, in some cases, excessive pressure had occasionally been applied, or too little chemical content was deposited (pair 1 younger mark), resulting in marks that were not recovered or that do not show clear ridge detail. Figure S2 reports the optical images of the natural sets showing instances of sub-optimal fingermark deposition. 

Generally, the separation of the ridge pattern was successful, and the number of older marks that could not be separated was lower than the groomed set. However, some additional observations need to be made. Older fingermark ridge patterns could not be separated for pair 4 (14 d vs. 3 h) and pair 5 (46 d vs. 3 h). However, as in the case of groomed pairs, the ridge detail for the marks of 26, 37, and 46 days of age could be separated/recovered in other instances (pairs 7 and 8).

In the case of pair 1 (1 d vs. 3 h), it was the younger fingermark’s ridge pattern (3 h) that could not be recovered. However, 3 hr old fingermarks were successfully imaged for the other pairs in this set of impressions (pairs 2–5). For pair 6, the squared frame highlights an area of the separated older mark, which possibly also exhibits some level of overlapping with the younger mark. Pairs 7 and 8 are interesting in that the squared frames highlight the fact that “separating ions” are not necessarily molecules unique to one fingermark, but they can be present in both, though in very different abundances such that separation can still occur. For pairs 7 and 8, the marks at 26 and 37 days are still visible, and whilst separated from the 46-day-old mark, the 26-day-old mark exhibits an even better clarity of the ridge detail.

As for the groomed fingermarks (Figure 1), the ions *m*/*z* 340.399 and 368.429 could be putatively assigned the formulas of C_23_H_50_N^+^ (mass accuracy −2.35 ppm) and C_25_H_54_N^+^ (mass accuracy −3.8 ppm), respectively. The ion at *m*/*z* 326.379 is also a contaminant and has previously been detected and identified in fingermarks as the didecyldimethylammonium ion [20]. The ions at nominal *m*/*z* 523 were suspected to be the external contaminant previously detected [21,22] of formula C_36_H_76_N^+^. However, the relative error is too high to permit this assignment (>20 ppm). A lipid maps search permitted the ion at *m*/*z* 381.247 to be putatively identified as a sterol lipid (M+H-H_2_O]+) of formula C_22_H_38_O_4_S (mass accuracy −2.8 ppm). The ion at *m*/*z* 415.15 could be assigned to another sodiated lysophosphatidic acid derivative (LPA 14:3;O) of formula C_17_H_29_O_8_PNa; however, the relative error of −12.5 ppm casts stronger doubts on this ion’s identity. The lipid maps search did not allow any other univocal putative identification for any of the remaining separating ions shown in Figure 2, and MS/MS experiments are necessary to establish their identity. 

**Figure 3:**
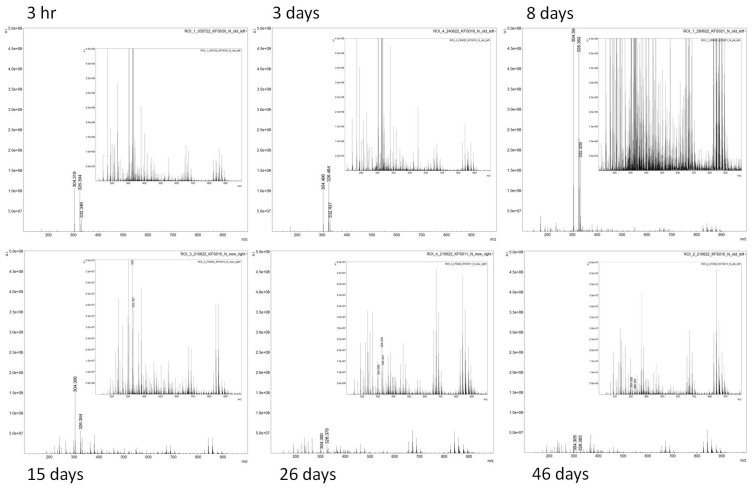
Representative mass spectra extracted from the data used to generate the images in Figure 2 by drawing regions of interest in the outermost left (older) and outermost right (younger) sides of the ion images, where the fingermarks do not overlap. The intensity is normalized to the total-ion-chromatogram for each spectrum. The two major ions at *m*/*z* 304 and 326, observed with the highest relative abundance in the younger fingermarks (the first two ions labelled in the spectra), have previously been identified as dodecylbenzyldimethylammonium and didecyldimethylammonium ions, respectively, and are common antibacterial products used, e.g., in hand-sanitizers [20,21,22].

Regions of interest were drawn from the natural set of marks from the non-overlapping areas; spectral variability reflecting variable molecular composition could be observed in the mass range investigated even by manual inspection (Figure 3), either in terms of absence/presence of signals or in terms of the relative intensity. 

As expected, the spectral profiles change dramatically with the age of the mark. Visually, the spectral profiles of marks of 3 h, 3 days, and 8 days of age differ significant from those with ages of 15 days, 26 days, and 46 days. In particular, the lower molecular range exhibits signals of higher intensity, as highlighted by the zoomed in spectra (for example for the 3h and 3 days vs. 8 days), possibly reflecting the breakdown/degradation of complex lipids with time. Though more donors and replicates would strengthen the reliability of this observation in a follow up study, this spectral variability explains the fingermark image separation achieved. The zoomed in spectra for the marks at 15, 26, and 46 days have a different (higher relative intensity) y scale compared to the other marks to better highlight any differences between these older marks; the ions at nominal *m*/*z* 304, 326, and 332 decrease in intensity as the age increases. The ions at *m*/*z* 304 and 332 are exogenous species and have been putatively identified as dodecylbenzyldimethylammonium (nominal *m*/*z* 304) with two extra CH_2_ units, respectively, also previously detected in fingermarks [20]. As reported earlier in this paper, the ion at nominal *m/z* 326 is also a contaminant and has previously been detected and/or identified in fingermarks as didecyldimethylammonium ion [21,22]. These ions are common species found in toiletries (e.g., hair gels) and antibacterial products, such as hand sanitizers. They are observed for all marks up to 46 days of age. Their constant presence in all the marks examined indicate these are persistent substances and can be used to obtain MALDI molecular images of fingermarks recovered at crime scenes that are not accessed immediately (up to 46 days of age in this study) due to their high ionisation efficiency. The mass spectral profiles of the 26- and 46-day-old marks appear to exhibit a higher ion population in the higher mass range (*m*/*z* 690–900) compared to the 15-day-old mark. Again, this is possibly reflecting the breakdown/degradation of high molecular weight compounds (beyond the mass range measured in this study) with time.

**Figure 4:**
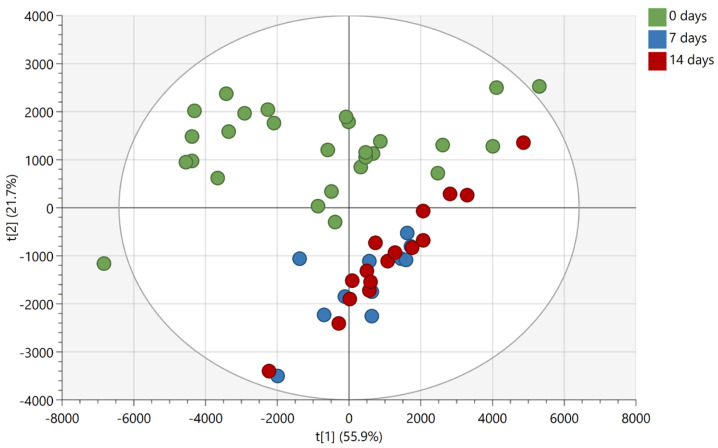
PCA score plot (R^2^X(cum) = 0.906; Q^2^(cum) = 0.815) of 0-, 7-, and 14-days-old fingermarks deposited by the same person on aluminium slides. The ellipse indicates a Hotelling’s T^2^ of 95%.

Based on the findings reported in Figure 2 and Figure 3, another experiment was conducted whereby the same donor deposited non-overlapping natural fingermarks, which were aged for 0, 7, and 14 days, matrix-spray-coated under the same conditions as for the overlapping fingermarks and subjected to manual firing of the laser in discrete locations (MALDI MS Profiling (MALDI MSP)). The 7- and 14-days-aging points for the chemical profiling were selected based on the more dramatic difference in spectral profiles observed for the overlapping fingermarks of 8 and 15 days of age (Figure 3). Mass spectra were subsequently processed for submission to multivariate statistical analysis. Principle component analysis (PCA) shows clear separation between fresh marks (0 days) and marks of older age points (7 and 14 days) (Figure 4). 

In contrast, group overlap was observed between the 7- and 14-days-age points indicating that, overall, the compositional change of marks between 7 and 14 days old is not enough to permit differentiation between 1-week- and 2-weeks-old marks. Importantly, however, the inability under these experimental conditions to differentiate between 7- and 14-day-old marks does not mean that unique molecules could not be found, allowing a separation of the ridge pattern for these two age points.

**Figure 5:**
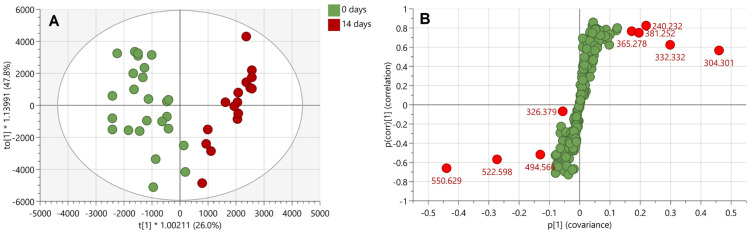
Multivariate statistical analysis of natural fingermark of 0 and 14 days of age. Panel A: OPLS-DA score plot (R^2^X(cum) = 0.829; R^2^Y(cum) = 0.833; Q^2^(cum) = 0.785) showing the discrimination between 0- and 14-days-old fingermarks deposited by the same person on aluminium slides. The ellipse indicates a Hotelling’s T^2^ of 95%. Panel B: S-plot highlighting the most discriminating ions in 0- and 14-days-old fingermarks in the OPLS-DA model. The x-axis shows the magnitude of the variables and their importance, while the y-axis indicates reliability; the closer to ±1, the more reliable. Red-labelled ions have a VIP score >1 (on a 95% confidence level).

A subsequent linear discriminant analysis was performed using OPLS-DA with a seven-fold cross validation on the marks of 0 and 14 days of age. The two 0-day old outliers observed in the PCA score plot (Figure 4) were excluded from data before performing the analysis. The OPLS-DA model could describe all included data (R^2^X(cum) = 0.829), showing clear separation of the two age groups (R^2^Y(cum) = 0.833) as well as robustness in the ability to classify and distinguish fresh from 14-days-old marks (Q^2^(cum) = 0.785) (Figure 5A). 

An S-plot based on the OPLS-DA model was used to reveal the most discriminant ions for the two age groups (Figure 5B). The most discriminating ions are *m*/*z* 304.301, 550.629, 332.332, and 522.598 with VIP scores of 6.3, 5.6, 3.9, and 3.7, respectively. Interestingly, despite the ion at *m*/*z* 326.379 displaying a high VIP score (4.5), suggesting a high contribution to the separation of the two groups of marks in the OPLS-DA model, the S-plot indicates instead a low reliability in the model, i.e., high risk of spurious correlation. Similarly to the ions at nominal *m*/*z* 304, 326 and 332, the ion at nominal *m*/*z* 551 is also an exogenous species and is putatively identified as the dimethyloctadecylammonium ion from previous published work [5,20,22]. In addition, the discriminating ion at *m*/*z* 499.566 may also putatively be identified as ditallowdimethylammonium ion, likely resulting from the use of personal and household products, as reported by Manier et al. [22]. This time the ion at nominal *m*/*z* 523 could be putatively identified as another ditallowdimethylammonium ion (C_36_H_76_N^+^, mass accuracy −1.9 ppm) belonging to the same family as the ion at *m*/*z* 494.566. Therefore, within this profiling experiment and based on the statistical analysis, the most age-discriminant ions are exogenous contaminants. However, as shown in Figure 2, endogenous compounds may also contribute to the separation between overlapping fingermarks.

In conclusion, this study indicates it is possible for this donor to separate overlapping fingermarks deposited at different times as a result of a different enough molecular composition. This result is supported especially by the use of natural marks (not artificially enriched). However, a multi-donor study is required to extend this observation to the general population. The output from this study is not in contrast with the work published by Gorka et al. [12]; intra-donor variability may still, as they conclude from their study, be very low. However, to separate overlapping fingermarks, even just one molecule per mark out of the thousands detected is needed to be unique to obtain a separate image of the two impressions. In the future, this capability will be very useful when supported by a method that definitively established the age of a fingermark to establish *(i)* one person has deposited both overlapping marks, *(ii)* the identity of the individual (through the separated ridge detail obtained), and *(iii)* the time at which the scene was accessed on both occasions (in the case of two overlapping fingermarks) to establish legitimate access.

## 3. Materials and Methods

### 3.1. Materials

Trifluoroacetic acid (TFA), α-cyano-4-hydroxycinnamic acid (α-CHCA) was purchased from Sigma Aldrich (Poole, UK). Acetonitrile (ACN), acetone, and methanol were purchased from Fisher Scientific (Loughborough, UK). The double-sided conductive carbon tape was obtained from TAAB (Aldermaston, UK). TLC sheets were purchased from (Merck, UK).

### 3.2. Methods

#### 3.2.1. Instrument and Instrumental Conditions

All MALDI MS spectrometric analyses were carried out using the Waters MALDI QTOF Synapt G2 HDMS instrument (Waters Corporation, Manchester, UK). Data acquisition was performed within the *m*/*z* range of 100–1000 in positive sensitivity mode with a scan time of 1 s after calibrating the instrument with a saturated solution of red phosphorus in ACN. The Nd:YAG laser repetition rate was set to 1 kHz and laser power to 250 a.u. for all analyses. MALDI MS Images were acquired at a spatial resolution of 100 µm × 100 µm. For chemical analysis, data were acquired from three to four (random) areas for each fingermark using a circular shutting pattern consisting of approximately 100 shots. 

#### 3.2.2. Data Processing of MALDI MS Data

MALDI MS fingermark images of the 4500 most intense ion signals were generated using the HDI software (v. 1.6, Waters Corp., UK) using a mass window of 0.02 Da. Visual inspection of those images led to the selection of the *m*/*z* ions that produced the best separation of the ridge pattern. MS spectra from each of the fingermark pairs were obtained through the selection of regions of interest in the outermost left and right sides of the ion images, respectively, where the fingermarks do not overlap.

#### 3.2.3. Preparation of Fingermarks Samples for MALDI MSI

TLC sheets were preliminarily prepared, removing the silica by soaking them in methanol and sonicating for 15 min. Upon wiping the residual silica with acetone, they were cut into glass slide sizes and used as the fingermark deposition surfaces. On eight of these resulting separate aluminium sheets, a groomed fingermark (deposited without washing hands at any particular time before the deposition but after rubbing the fingertip on forehead and cheeks) was deposited by the same donor and allowed to age for a definite time at ambient office conditions. Subsequently, the same donor deposited a second groomed fingermark partially overlapping the first, and the two impressions were allowed to age further for a definite time period. Specifically, eight pairs of different age overlapping fingermarks were deposited, as summarised in Table 1, where sets 1–5 intended to investigate the separation between old and fresh fingermarks and sets 6–8 the separation between old fingermarks (but of different age).

An additional set of eight pairs of overlapping natural fingermarks (deposited without prior preparation of the fingertip, i.e., without washing hands or touching skin at any particular time before the deposition) were also prepared and aged in the same way. Matrix deposition was optimised, as described in Section 3.2.5, and the aluminum sheets were then mounted onto the Synapt MALDI target plate using double-sided conductive carbon tape. All samples were imaged by MALDI MS immediately after deposition of the matrix. The time delay between the deposition of the youngest fingermark and the start of MALDI MS analysis was 20–30 min (this time delay is included in the indicated ages of the fingermarks).

#### 3.2.4. Preparation of Fingermark Samples for Chemical Analysis

On seven separate aluminium slides, natural smudged fingermarks were deposited by the same person. Three of the fingermarks were allowed to age for seven days, while the remaining four were aged for fourteen days, all at ambient office conditions. Another fresh (25–107 min) smudged fingermark (natural) was then deposited by the person on the same aluminium sheet (one per slide for a total of seven) but without overlapping the first fingermark. The matrix was then sprayed, as described in Section 3.2.5, and the aluminium slide was mounted onto the MALDI sample plate using double-sided conductive carbon tape. The samples were analysed by MALDI MS immediately after deposition of the matrix. Three to four technical replicates were measured for each fingermark.

#### 3.2.5. Matrix Application

α-CHCA matrix was prepared at a concentration of 5 mg/mL in 70:30 ACN:0.5% TFA_aq_. The matrix was sprayed using the HTX M3+ automated sprayer (HTX Technologies, Chapel Hill, NC, US) and deposited onto the sample in four layers (no dry time between passes) with an alternating vertical and horizontal spraying pattern and a track spacing of 4 mm. Flow rate, sprayer velocity, pressure, and temperature were set at 0.1 mL/min, 1200 mm/min, 10 psi, and 75 °C, respectively.

#### 3.2.6. Multivariate Analysis

The MALDI MS data from the chemical analysis of the aged (non-overlapping) fingermarks were transformed from continuum to centroid spectra in MassLynx (Waters Corp.) using automatic peak detection with subtraction of background (polynomial order 15, below curve 10%, tolerance 0.010). All spectra were then imported into SpecAlign (v. 2.4.1; Cartwright Group, PTCL, University of Oxford) for peak alignment. Baseline was subtracted (window size 5), and the data were de-noised (threshold 0.5) and normalized to TIC (*m*/*z* range 100–1000). The spectra were then aligned to an average spectrum using the PAFFT algorithm (minimum segment size: 449 points; max shift: 20; scale: 1; reference: 0). Peaks were selected automatically (baseline cutoff = 0.5; window = 1; height ratio = 1.5). Peaks with a maximum intensity less than 10,000 were manually removed. Peaks corresponding to isotopes and matrix adducts were also removed. The processed data, containing a total of 194 ion peaks, was imported into SIMCA (v. 16.0.1; Sartorius Stedim Data Analytics AB, Göttingen, Germany) for multivariate statistical analysis. Pareto scaling was applied before principal component analysis (PCA) and orthogonal partial least-squares discriminant analysis (OPLS-DA). The unsupervised PCA served as a quick visualization of data, while the supervised OPLS-DA was used to differentiate between 0- and 14-days-old fingermarks and to identify ions important for the group separation. Hotelling’s T^2^ with a significance level of 0.05 were used to identify potential outliers. Three components were used for the OPLS-DA model. The parameters Q^2^, R^2^X, R^2^Y (X being the matrix of ion features, and Y the matrix of the 0- and 14-day groups) were used to evaluate the performance of the model. Q^2^ was obtained by seven-fold cross validation and explains the predictability of the model, whereas R^2^ explains how well the model fit the data. A Q^2^ score >0.4 and an R^2^ > 0.5 is considered to indicate a robust model.

## Figures and Tables

**Table 1:** Partially overlapping groomed fingermark pair sets prepared, aged at room temperature, and analysed by MALDI MSI. (Note “d” stands for days).

Pair Set	Fingermark 1 Age (Older)	Fingermark 2 Age (Younger)
1	1 d	1 h
2	3 d	1 h
3	8 d	3 h
4	14 d	1 h
5	46 d	1.5 h
6	46 d	15 d
7	46 d	26 d
8	46 d	37 d

**Table 2:** Natural partially overlapping same donor’s fingermark pair sets aged under the same environmental conditions as for the groomed mark pairs (but with slightly different durations in time) and analysed by MALDI MSI. (Note "d" stands for days).

Pair Set	Fingermark 1 Age (Older)	Fingermak 2 Age (Younger)
1	1 d	3 h
2	3 d	3 h
3	8 d	5 h
4	14 d	3 h
5	46 d	3 h
6	46 d	15 d
7	46 d	26 d
8	46 d	37 d

## Data Availability

Raw data will be uploaded on the SHURA repository, upon acceptance of the manuscript.

## Supplementary Materials

The following supporting information can be downloaded at: https://www.mdpi.com/article/10.3390/molecules28062763/s1. Figure S1. Optical images of eight pairs of partially overlapping same-donor groomed fingermarks on aluminium slides. Majority of the images show excessive contact pressure during fingermark deposition, likely responsible for lack of separation and/or of suitable ridge detail quality after analysis by MALDI MSI. Figure S2. Optical images of eight pairs of partially overlapping same-donor natural fingermarks on aluminium slides. Some of the images show excessive contact pressure during fingermark deposition, likely responsible for lack of suitable ridge detail quality after analysis by MALDI MSI.

## Sample Availability

Samples of the compounds used in the present study are no longer available after being analysed.
