# Supplementary figures and images for: MALDI MSI Separation of Same Donor’s Fingermarks Based on Time of Deposition—A Proof-of-Concept Study
Source: Molecules. 2023 Mar 19;28(6):2763. doi: 10.3390/molecules28062763 (PMC10054356; doi:10.3390/molecules28062763)

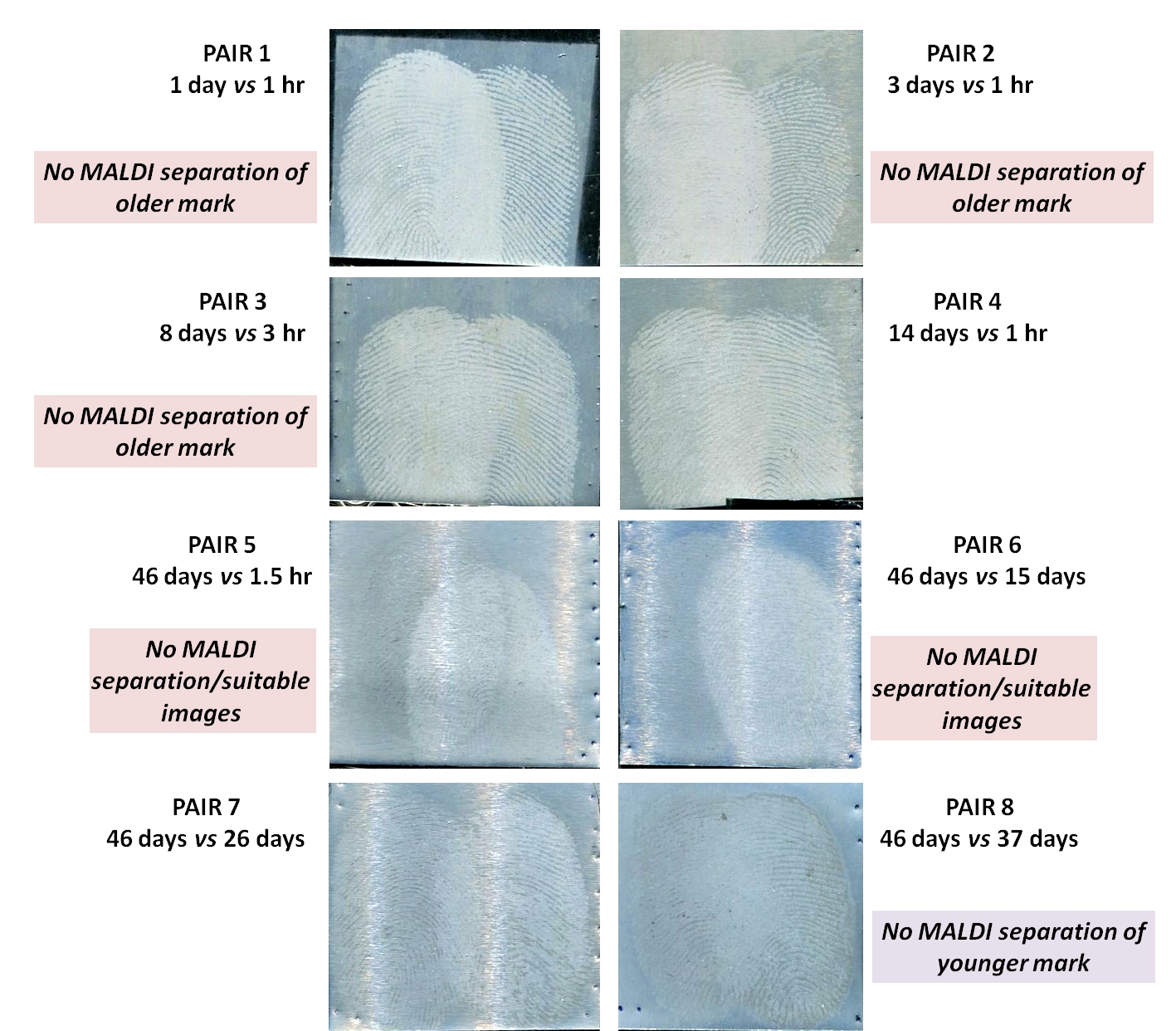

Supplement: Supplementary file 1 [file molecules-28-02763-s001.zip › Supplementary Figures S1 and S2/Figure S1.jpg]

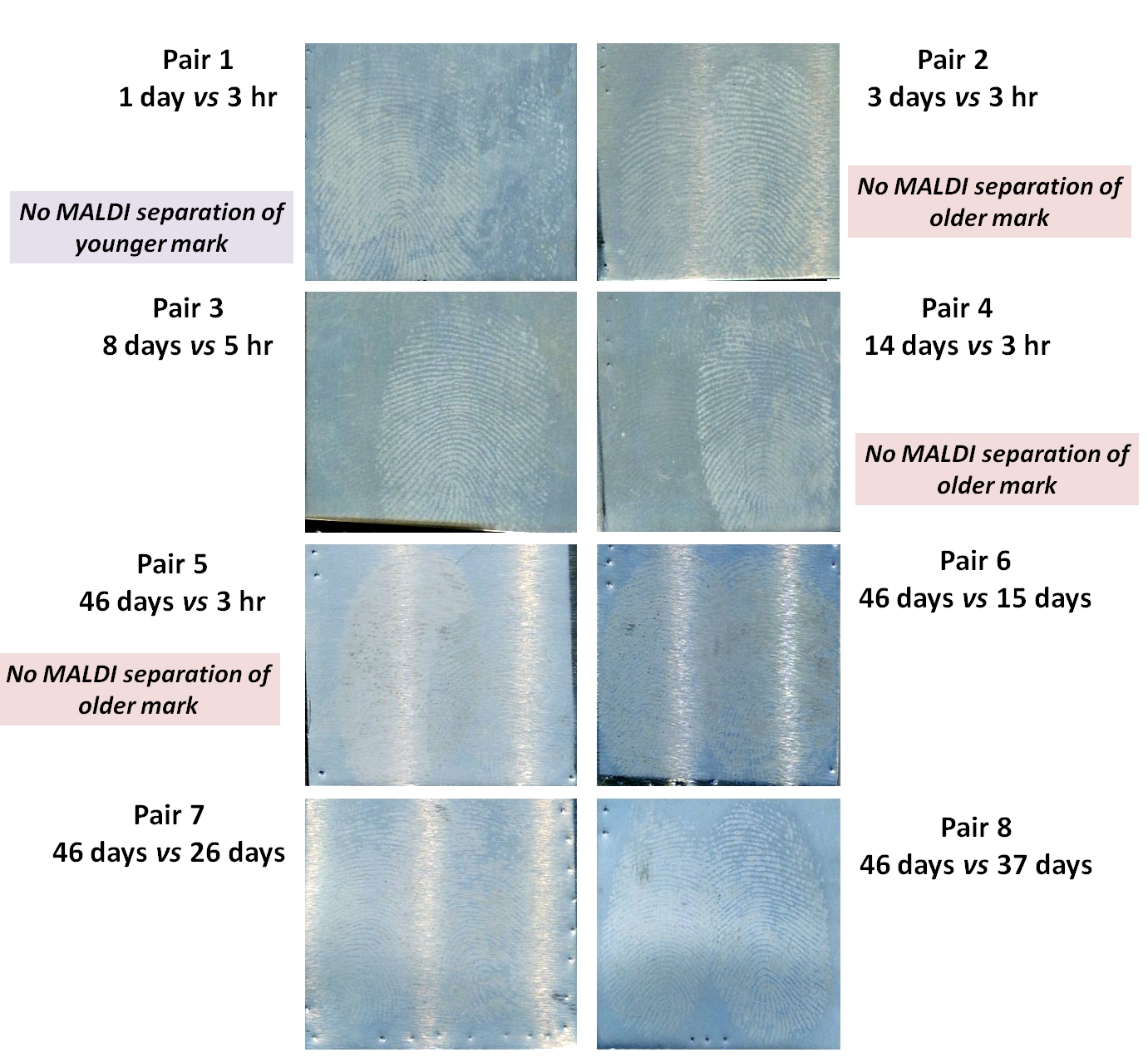

Supplement: Supplementary file 1 [file molecules-28-02763-s001.zip › Supplementary Figures S1 and S2/Figure S2.jpg]
